# Infant HIV testing at birth using point-of-care and conventional HIV DNA PCR: an implementation feasibility pilot study in Kenya

**DOI:** 10.1186/s40814-019-0402-0

**Published:** 2019-01-25

**Authors:** Matthew R. Sandbulte, Brad J. Gautney, May Maloba, Catherine Wexler, Melinda Brown, Natabhona Mabachi, Kathy Goggin, Raphael Lwembe, Niaman Nazir, Thomas A. Odeny, Sarah Finocchario-Kessler

**Affiliations:** 1 Department of Family Medicine, University of Kansas Medical Center, Kansas City, KS USA; 2 Global Health Innovations, Dallas, TX USA; 3 Global Health Innovations, Nairobi, Kenya; 4 Health Services and Outcomes Research, Children’s Mercy Hospitals and Clinics, Kansas City, MO USA; 5 University of Missouri–Kansas City School of Medicine, Kansas City, MO USA; 6 Centre for Virus Research, Kenya Medical Research Institute, Nairobi, Kenya; 7 Department of Preventive Medicine and Public Health, University of Kansas Medical Center, Kansas City, KS USA; 8 Center for Microbiology Research, Kenya Medical Research Institute, Nairobi, Kenya

**Keywords:** HIV, Early infant diagnosis, Point-of-care testing, Birth testing, Implementation

## Abstract

**Background:**

Infant HIV diagnosis by HIV DNA polymerase chain reaction (PCR) testing at the standard 6 weeks of age is often late to mitigate the mortality peak that occurs in HIV positive infants’ first 2–3 months of life. Kenya recently revised their early infant diagnosis (EID) guidelines to include HIV DNA PCR testing at birth (pilot only), 6 weeks, 6 months, and 12 months postnatal and a final 18-month antibody test. The World Health Organization (WHO) approved point-of-care (POC) diagnostic platforms for infant HIV testing in resource-limited countries that could simplify logistics and expedite infant diagnosis. Sustainable scale-up and optimal utility in Kenya and other high-prevalence countries depend on robust implementation studies in diverse clinical settings.

**Methods:**

We will pilot the implementation of birth testing by HIV DNA PCR, as well as two POC testing systems (Xpert HIV-1 Qual [Xpert] and Alere q HIV-1/2 Detect [Alere q]), on specimens collected from Kenyan infants at birth (0 to 2 weeks) and 6 weeks (4 to < 24 weeks) postnatal. The formative phase will inform optimal implementation of birth testing and two POC testing technologies. Qualitative interviews with stakeholders (providers, parents of HIV-exposed infants, and community members) will assess attitudes, barriers, and recommendations to optimize implementation at their respective sites. A non-blinded pilot study at four Kenyan hospitals (*n* = 2 Xpert, *n* = 2 Alere q platforms) will evaluate infant HIV POC testing compared with standard of care HIV DNA PCR testing in both the birth and 6-week windows. Objectives of the pilot are to assess uptake, efficiency, quality, implementation variables, user experiences of birth testing with both POC testing systems or with HIV DNA PCR, and costs.

**Discussion:**

This study will generate data on the clinical impact and feasibility of adding HIV testing at birth utilizing POC and traditional PCR HIV testing strategies in resource-limited settings. Data from this pilot will inform the optimal implementation of Kenya’s birth testing guidelines and of POC testing systems for the improvement of EID outcomes.

**Trial registration:**

ClinicalTrials.gov, NCT03435887. Registered 26 February 2018.

**Electronic supplementary material:**

The online version of this article (10.1186/s40814-019-0402-0) contains supplementary material, which is available to authorized users.

## Background

In 2015, an estimated 110,000 new pediatric human immunodeficiency virus (HIV) infections occurred in the 21 Global Plan priority countries [1]. Infant survival and the success of antiretroviral therapy (ART) treatment of HIV-positive infants are associated with early infant age at initiation [2]; thus, early HIV testing, diagnosis, and prompt ART initiation are crucial. In 2015, an estimated 6600 children were infected with HIV in Kenya, a 55% decrease since 2009, but the country’s rate of HIV-exposed infants receiving a routine HIV DNA polymerase chain reaction (PCR) test by 2 months of age has stagnated near 50% since 2011 [3, 4].

One barrier to successful early infant diagnosis (EID) is the waiting time for test results. The sequence of EID services involves multiple time-sensitive steps requiring coordination between medical provider, diagnostic laboratory, and mother/caregiver (“mother”). The standard EID algorithm in Kenya includes mother acceptance of infant testing; specimen collection, transport, and laboratory analysis; communication of results to healthcare providers and caregivers; and linkage to care, including opportunistic infection prophylaxis for all HIV-exposed infants and ART for HIV-positive infants [5]. All steps are susceptible to delays and patient loss to follow-up [5–7]. Traveling long distances for clinical care adds logistical and resource barriers to successful HIV treatment [8].

Innovative strategies with potential to accelerate the delivery of appropriate care to HIV-exposed infants include launching EID at birth rather than the standard 6 weeks of age and streamlining the EID cascade [9]. While these approaches are promising, resource constraints for all stakeholders in EID services (e.g., providers, laboratories, and mothers) require consideration. Adding a testing time point at or near birth, with standard-of-care (SOC) or point-of-care (POC) technology, would likely require more clinical and laboratory workload and equipment capacity [10], and potentially more travel to the clinic by mothers and infants. Conventional testing of HIV-exposed infants has not been initiated until 6 weeks of age because DNA PCR test sensitivity is lower in newborns and neonates (68% and 88% at birth and 4 weeks [11]), intrapartum (IP) HIV infections are not detectable immediately after birth, and the 6-week time point coincides with immunization visits. HIV-infected infants first tested around 6 weeks often are initiated on ART at median ages of 16.0 to 24.5 weeks [12–15], which is too late to mitigate the early peak in mortality around 8–12 weeks of age [16]. In Kenya, 61% of pregnant women deliver in a health care facility and 20% of the infants delivered at home receive a postnatal checkup within 1 week of birth [17]; therefore, testing performed at birth or first clinical contact could accelerate ART initiation in HIV intrauterine-infected infants. Recent recommendations by the WHO and the Kenya Ministry of Health [18, 19] reflect a consensus that birth HIV testing could improve outcomes for HIV-infected infants, even with lower test sensitivity on newborn samples [20, 21]. Kenya’s guidelines call for piloting birth HIV DNA PCR testing of HIV-exposed infants, in addition to further HIV DNA PCR tests at 6 weeks, 6 months, and 12 months. However, pilot data for birth testing (0–2 weeks) is needed prior to national rollout.

In 2016, the WHO prequalified two point-of-care (POC) diagnostic tests for detection of HIV RNA in whole blood samples (Xpert HIV-1 Qual for GeneXpert instruments, manufactured by Cepheid AB; Alere q HIV-1/2 Detect manufactured by Abbott). Both tests are cartridge-based, real-time reverse transcription PCR qualitative assays performed with benchtop instruments that are suitable for operation in the clinic setting and do not require extensive technical training [22, 23]. The initial published evaluations of the Xpert HIV-1 Qual or Alere q HIV-1/2 tests performance were conducted by diagnosticians in the laboratory setting [22–25], showing good sensitivity (93.3–98.7%) and specificity (100%) with infant samples. By definition, the POC test systems provide optimum value in expediting EID if they are performed in the clinic setting, by clinical personnel. Field studies of both the Xpert HIV-1 Qual [26, 27] and Alere q HIV-1/2 [27–29] were performed in clinical care facilities, by medical caregivers. Sensitivity and specificity values were similar to those reported from the laboratory-based qualification studies, but in some instances the field studies produced higher rates of test error [26, 29]. Recent studies with large numbers of HIV-positive newborn infants show close concordance between SOC tests and both of these POC technologies [26, 27]. While analytic performance profiles of these POC technologies have been characterized in field studies, sustainable scale-up and optimal utility in Kenya and other resource-constrained countries requires robust implementation studies in diverse clinical settings. Currently, there is a lack of data to guide strategic implementation of the birth testing timepoint or the Xpert and Alere q POC testing platforms in Kenya. Here, we report the protocol for a pilot study to assess feasibility and preliminary impact of introducing birth testing as an addition to EID services, and to evaluate the two POC platforms in parallel with laboratory-based HIV DNA PCR.

### Objectives

1. Assess acceptability and uptake of birth testing and both prequalified POC testing platforms in Kenyan clinical settings.
2. Collect pilot data quantifying the efficiency and completeness of EID services augmented with birth and POC testing.
3. Capture pilot data tracking the reliability/performance of both POC platforms for EID services.
4. Engage EID providers in interviews to assess user satisfaction with birth and POC testing and gather feedback on implementation barriers and facilitators.
5. Compute costs of implementing POC testing platforms to fulfill Kenyan EID guidelines.

## Methods

### Study design

In this feasibility study, the new birth EID testing timepoint and two POC infant diagnostic platforms will be implemented on a pilot scale in Kenya. Xpert and Alere q HIV POC platforms will be introduced at four Kenyan government hospitals, for birth and 6-week testing time points. This 18-month study was initiated as a supplement to a parent study (R01HD076673) implementing and evaluating the HIV infant tracking system (HITSystem) [30]. The four hospitals designated as study sites are government hospitals with medium to high patient volume, and geographic variation: two in Kisumu County, one in Nakuru County, and one in Mombasa County. Estimated rates of mother to child transmission of HIV in these three counties in 2015 were 20%, 5%, and 18%, respectively [31]. Phase 1 is a 6-month formative phase in which we will conduct qualitative interviews with parents, providers, and community members to assess stakeholder attitudes toward birth and POC testing and barriers to acceptance or uptake. Feedback will be used to refine operational plans with regard to feasibility and acceptability. Phase 2 is a 12-month feasibility pilot of the two new POC testing strategies. The 12-month implementation period allows time for providers to adopt the new practices and to monitor any fluctuations in uptake or acceptability during this extended period. POC HIV testing will be conducted in parallel with standard laboratory-based HIV DNA PCR, targeting sample collection at birth (0 to 2 weeks) and at 6 weeks (4 to < 24 weeks).

### Phase 1: formative research

Phase 1 seeks to gain information from key stakeholders that will guide operational planning to optimize uptake, acceptability, and feasibility of services at each study site. Approximately *n* = 25 formative interviews with parents, providers, and community members will be conducted at each site; 100 interviews total. Interviews with parents (HIV-positive mothers with prior EID experience and their male partners if available and mother has already disclosed HIV status, *n* = 10 per site) will focus on the impact for the infant and family (confidentiality/disclosure, acceptability of timing of testing and results, and partner support). Interviews with providers who would be involved with POC testing at each site (PMTCT, maternity, and maternal and child health (MCH) nurses; mentor mothers; laboratory technicians; and ART clinicians; approximately *n* = 5 per site) will highlight issues of training, site-specific logistics, patient preparation and counseling, and personnel and resource considerations for implementation. We will also conduct interviews with community members (community health workers, traditional birth attendants, and respected community and religious leaders; approximately *n* = 10 per site) in one rural community within the catchment area of each hospital to elicit attitudes and suggestions regarding the potential for periodic POC HIV testing in hard to access communities. Informed consent will be obtained prior to conducting the interviews in a private setting. Participants will receive 500 Kenyan Shillings (approximately USD $5.00) to compensate for travel and time.

#### Analysis

Interviews will be audio recorded for subsequent transcription, translation, coding, and analysis. Transcripts will be coded independently by two study team members to identify a priori and emergent themes. Discrepancies in coding will be resolved by group consensus. We will develop a codebook with typical exemplars for each theme, noting the frequency and distribution of themes within the larger topic areas. The study team will review themes to inform the POC pilot (phase 2).

### Phase 2: intervention

#### Study design

**Fig. 1:**
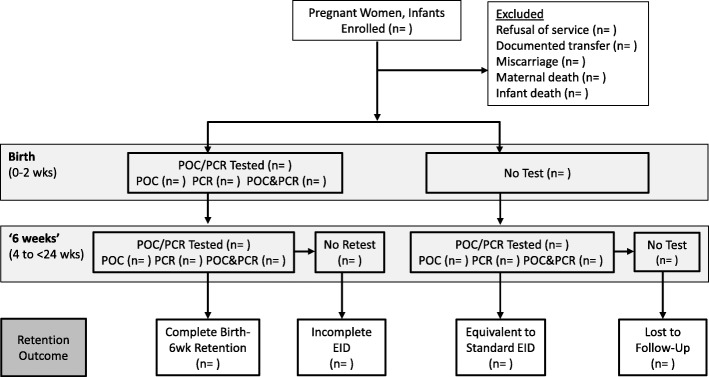
Patient flow during evaluation of infant HIV testing strategies. The study will track engagement of enrolled mother-infant pairs in point-of-care (POC) and standard of care HIV DNA polymerase chain reaction (PCR) test technologies through the birth and 6-week testing windows

We will pilot the implementation of POC and birth testing at four government hospitals over a 12 month period. The four study sites will be randomized, using a random number generator, to pilot one of the two POC systems (Xpert HIV-1 Qual for HIV RNA or Alere q HIV-1/2 Detect). SOC diagnostic testing (HIV DNA PCR test on DBS samples) will be conducted in parallel at all study sites, including the newly introduced birth testing time point. Patient flow in the pilot is diagrammed in Fig. 1.

#### Participant recruitment, eligibility, and consent

HIV-positive pregnant women or mothers of HIV-exposed infants engaged in care at study hospitals will be invited to participate in the pilot. Women can be enrolled either through PMTCT, during pregnancy; through maternity, at delivery; or through the MCH department, at their first postnatal infant visit. Women must be at least 18 years of age and their HIV-exposed infant less than 24 weeks of age to be eligible for the study. Pregnant women/mothers will be informed by trained research or clinical staff of the purpose of the research, the potential benefits, and the risks. They will also be counseled on expectations for POC testing at the time of delivery or within 2 weeks if delivery does not occur in the hospital, and the importance of returning for the birth PCR results and retesting at 6 weeks. Those agreeing to participate will provide written informed consent prior to enrollment in the study (Additional file 1). Clinical staff will promote participant retention and complete follow-up by continued outreach to enrolled mothers at ANC and MCH appointments. Infants of mothers who decline participation in the study will receive the on-site standard of care (SOC) EID (laboratory-based HIV DNA PCR testing at 6 weeks). The study protocol complies with the Helsinki Declaration and was reviewed and approved by the Institutional Review Boards at the Kenya Medical Research Institute (protocol KEMRI/SERU/CVR/018/3390) and the University of Kansas Medical Center (protocol # 00140399). A data monitoring committee was not required because the interventions are minimally invasive diagnostic strategies posing no elevated risks of severe outcomes. Protocol modifications will be communicated to facility personnel by research team site coordinators.

#### Pilot POC intervention

##### Personnel training

Study staff will be trained in procedures for informed consent and study enrollment and the protection of patient confidentiality. The hospital maternity staff will be trained to conduct a heel stick and collect two samples at each time point (DBS and whole blood). A half-day of training will be provided to EID staff on the equipment, cartridges, and procedures used to conduct POC tests at their hospital. Study coordinators at each site will arrange periodic refresher training and new personnel training as rotation of staff occurs.

##### Birth testing

For all infants presenting for birth testing (within 2 weeks of age), the designated staff at each facility will collect two blood samples. A whole blood sample for POC testing (Xpert HIV-1 Qual or Alere q HIV-1/2 Detect) will be processed on-site by healthcare providers using test cartridges and POC machine. POC test results will be recorded in study logs and printout results from each of the POC machines will be attached to the infant’s clinical file (see *Data collection*, below). Mothers will be notified of the POC test result on the same day as testing or before infant’s hospital discharge (within 24 h). The second sample will follow the established SOC procedures for HIV DNA PCR testing: a dried blood spot (DBS) sample will be sent by courier to the hospital’s designated central laboratory for standard HIV DNA PCR. Results of HIV DNA PCR processed in the central laboratory will be recorded on paper forms and returned to the hospital by courier service. When the HIV DNA PCR result is received at the hospital, the mother will be called to return for result notification and counseling. If the infant has an HIV-positive test result (by either POC or PCR), the mother and infant will be linked to the Comprehensive Care Center (CCC) for same-day ART initiation. HIV-positive infants identified through this study will receive additional clinical monitoring (ART adherence, viral suppression, and immune system strength) through 24 months of age. HIV-negative infants will be scheduled to return to the hospital for six-week postnatal appointments and repeat HIV testing (HIV DNA PCR and POC).

##### Six-week testing

All infants who present for care at 4 to < 24 weeks of age, not previously diagnosed HIV-positive, will be eligible for 6-week testing. The same sequence of DBS and whole blood sample collection, DBS shipment for processing off-site, POC processing in clinic, result notification, and ART initiation (for HIV-positive infants) will be conducted as at birth. The optimal test sequence of an HIV-negative infant will be complete when HIV results at birth and 6 weeks postnatal have been provided to mothers.

##### Discordant results

In the case of discordant POC/HIV DNA PCR test results at birth or at 6-weeks, the infant will be initiated on ART based on the positive result from either method. A second, confirmatory sample will be collected and processed using both the POC and HIV DNA PCR procedures. A third sample will also be sent to the national reference lab for HIV DNA PCR processing. The infant will continue on ART until the results of the HIV DNA PCR test and the National Reference Lab results are available. ART continuation will be based on the HIV DNA PCR results.

##### ART adherence monitoring of HIV-positive infants

In order to monitor ART adherence and viral suppression, we will order quarterly CD4 and viral load (VL) tests for all HIV-positive infants until 24 months of age. At each time point, mothers of HIV-positive infants will take a brief survey, administered by the pediatric ART provider, regarding ART adherence and side effects. Since we anticipate that infants will be started on ART significantly earlier than in SOC, this increased monitoring is intended to characterize infants’ virologic and immunologic responses to early treatment, providing preliminary data to inform design of a larger-scale randomized trial.

##### Data collection

Using Epi Info software (US Centers for Disease Control and Prevention), site coordinators will collect and maintain a comprehensive electronic record of each infant’s relevant clinical data including date of birth, location of birth, POC sample collection and processing dates, POC test results, POC result notification to mothers, dates of SOC sample collection and return of results to the hospital, results of HIV DNA PCR test, mother notification of HIV DNA PCR results, and date of initiation of HIV-positive infants on ART. To ensure complete and accurate clinical data entry, site coordinators will periodically cross-check Epi Info files with various clinical records including ANC registers, maternity records, HIV-exposed infant registers, mother and infant clinical files, and hard copies of laboratory diagnostic results. Data pertaining specifically to treatment of HIV-positive infants (including ART initiation, OI prophylaxis, and viral load and CD4 monitoring through 24 months) will be accessed from HIV exposed infant registers, CCC records, and infant clinical files.

Study-specific data collection logs will track enrollment across varying departments, reasons for enrollment refusal, use of the POC machines including dates and times of sample collection and processing and the personnel processing the test, POC machine errors and their effect on clinical care, adverse events that occur as a result of the study, communication between clinical or study staff and participants, and hospital or national-level events that may impact clinical care and study operations. These logs will help gauge implementation feasibility and provider experience. The principle investigator and IRB-certified research staff will have access to the final trial dataset, with no contractual hindrances.

##### Experience of providers

Qualitative data will be collected on providers’ implementation experience. The study site coordinator at each hospital will conduct monthly meetings including department heads (PMTCT, Maternity, MCH, Laboratory, Outpatient Department, and CCC) and all providers engaged in study implementation. While meeting guides were developed using the Consolidated Framework for Implementation Research (CFIR) as a guide [32], questions will vary to ensure responsiveness to provider concerns and challenges expressed at different stages of the study. Topics of these discussions will include training and sensitization of personnel; stakeholder coordination; sensitization, enrollment, and satisfaction of mothers; deployment of POC testing; workflow optimization; management of the test cartridge supply stream; technical challenges; and context-specific solutions. The study team will review themes after each meeting to inform any required modifications to POC implementation (phase 2). Participants will complete an informed consent at the first meeting for permission to audio record the sessions for later transcription and analysis of the implementation experiences at each hospital.

##### Calculating costs of POC

Costs associated with each POC strategy will be collected and compared to the existing EID testing process. We will estimate costs from a donor or government perspective to calculate and compute costs of integrating each POC strategy into an existing system compared to HIV DNA PCR processed at central laboratories, the current gold standard for EID care. We will use standard procedures for intervention cost estimation [33].

#### Fidelity assurance procedures

Standard operating procedures (SOPs) were developed to standardize training, enrollment, and data collection. Personnel will be trained on the SOPs, which will be kept available at each site for convenient reference. Study management will provide supportive supervision visits quarterly to assess protocol adherence. The objectives of these visits include overseeing and assisting with formative interviews with key stakeholders, providing site-level training on study protocols to clinical and study staff, review of consent forms, and discussion of any challenges with the study team. Technical competence and compliance with POC SOPs will be evaluated by a checklist that includes maintenance of workspace, blood collection technique, operation of the machine, cartridge-loading technique, following machine prompts through to result reporting and archiving, biosafety and waste disposal procedures, and test documentation. Furthermore, the study team will hold a bi-weekly study call to update enrollment numbers, review study logs, and address any challenges or concerns with the consent process or study implementation. Site coordinators are required to report any adverse event immediately to the US- and Kenya-based principle investigators. Adverse events would include unintentional disclosure of HIV status or emotional distress related to study participation. Infant death is a non-study-related adverse event also reported to the IRB.

Performance and reliability of the POC tests will be monitored by sending every tenth specimen to the national reference laboratory for confirmatory testing by HIV DNA PCR. By agreement with the national POC technical working group, each infant sample with an HIV-positive POC or HIV DNA PCR test result will be followed up by collection of a second sample for confirmatory testing at the national reference laboratory.

#### Confidentiality

Study staff will be trained on the protection of patient confidentiality. Prior to the study, study site coordinators will meet with clinic staff and peer counselors to identify additional strategies appropriate to the local setting to improve participant confidentiality. Access to electronic data will be restricted to research staff directly involved in implementation and evaluation of the pilot. All electronic data will be de-identified and recorded through use of an identification number for each HIV-positive woman and her HIV-exposed infant. The link between participant name and identification number is known only by the site coordinator who conducts enrollment and by the provider who has an established care relationship with the participant. The record linking patient name and electronic ID will be securely stored in a locked office. De-identified data will be used for all analyses and results will be reported as aggregates and ratios.

#### Study outcomes

##### Acceptability and uptake

Key informant interviews will document attitudes, preferences, perceived benefits, and concerns from the perspective of stakeholders (HIV-positive mothers, EID providers, community members) (Table 1). Uptake of testing, by POC and SOC methods, will be quantified in terms of proportions of infants who are tested (have a documented specimen collection) in the birth and 6-week windows and the proportion of infants determined HIV-negative at birth who are presented again for retesting in the 6-week window.

**Table 1:** Implementation and feasibility measures

Variable	Measure (for both POC and HIV DNA PCR tests, unless specified)	EID stage(s)
Acceptability/uptake	Mothers’ views of birth testing impact on infant, family	
	Provider recommendations/concerns on implementation	
	Community member attitudes and recommendations	
	Proportion (%) infants with specimen collected	Birth, 6 weeks^a^
	Median infant age at specimen collection	Birth, 6 weeks
	% Birth-tested infants returned for 6-week retest	Sequential
Infant testing outcomes	% Tests with results returned	Birth, 6 weeks
	% Tests with mother notified of results	Birth, 6 weeks
	Median turn-around time (TAT), specimen to test result availability	Birth, 6 weeks
	Median infant age at result availability	Birth, 6 weeks
	Median TAT, result availability to mother notification of results	Birth, 6 weeks
	Median overall TAT, specimen to mother notification of results	Birth, 6 weeks
	Median infant age at mother notification of results	Birth, 6 weeks
	Median infant age at ART initiation (if diagnosed HIV+)	Birth, 6 weeks
	Complete retention: % mothers notified of birth and 6-week results	Sequential
POC platform performance	Number failed POC tests, GeneXpert and Alere q	Pooled
	Number POC missed opportunities due to machine breakdown	Pooled
	Number POC missed opportunities due to machine error	Pooled
	Number POC missed opportunities due to cartridge stockout	Pooled
	Concordance of POC results (each platform) with HIV DNA PCR	Birth, 6 weeks
Provider feedback	Acceptability and feasibility of implementing birth and POC testing	
	Benefits and challenges of birth and POC testing for patients	
Costs of POC implementation	Acquisition of GeneXpert and Alere q machines	
	Acquisition of accessory equipment	
	Site-specific training, secure storage	
	Acquisition of test cartridges	
	Maintenance and repair of machines	

^a^Birth testing is denoted when specimen is collected at 0–2 weeks of age. “Six-week” testing is denoted when specimen is collected at 4 to < 24 weeks of age

##### Infant testing outcomes

The completeness and efficiency of fulfillment of Kenyan EID testing guidelines upon implementation of birth and POC testing at study sites will be measured (Table 1). Measures of completeness will include proportions of birth and 6-week tests with results returned and with mothers notified of results. Additionally, we will track “complete retention,” the proportion of mothers who are notified of results from specimens collected in both the birth and 6-week windows. Measures of efficiency include turnaround time (TAT) associated with key steps in POC or HIV DNA PCR-based diagnosis: TAT from specimen collection to result availability, TAT from result availability to mother notification of results, and overall TAT from specimen collection to mother notification. Infant age at completion of each step of diagnosis (and at ART initiation if testing HIV-positive) will be further measures of EID efficiency that imply clinical timeliness of the specific EID steps.

##### POC platform performance

Assessment of both the Xpert and Alere q HIV POC platforms will include evaluation of testing reliability (Table 1). Measures to be tracked include number of failed tests (and wasted cartridges), and number of missed opportunities to engage infants with POC testing due to documented machine breakdown, machine error, or cartridge stockout. The anticipated number of HIV-positive infants identified is too small to support robust sensitivity or specificity calculations, but we will track concordance between test results from each POC platform and the corresponding HIV DNA PCR results.

##### EID provider feedback

Provider interviews conducted during the pilot study will solicit provider feedback on challenges faced in implementing birth and POC testing, including matters of personnel training, optimizing workflow, engagement of mothers, management of cartridge supply, and technical challenges. Providers’ views of the benefits and risks of birth and POC testing for patients will be recorded.

##### Costs of POC testing implementation

Cost measures to be tracked and analyzed for each of the POC test platforms include up-front purchase of machines and accessory equipment; site-specific training and secure equipment storage; purchase of test cartridges, including delivery and customs fees; and machine repair.

### Statistical analysis

This study is designed to gather preliminary outcome data to inform implementation guidance and pilot data for a subsequent larger study. We anticipate mean monthly enrollment per study site of approximately 15 eligible participants, yielding an estimated total enrollment of 720 mother-infant pairs by the end of the 12-month intervention phase. Reductions in study participation are expected to result from documented transfer to other facilities, loss to follow-up, miscarriage, and infant mortality prior to testing. Nonetheless, we anticipate ample power to describe and compare outcomes of birth testing and between POC and PCR testing strategies to estimate effect sizes and an intracluster correlation coefficient (ICC) to inform future sample size and power calculations.

Categorical variables will be presented as proportions, and continuous variables as means (SD) or medians (IQR) depending on the distribution of data. Quantitative measures compared across POC platforms or between POC and HIV DNA PCR testing methods will be analyzed using parametric and non-parametric (Exact or Wilcox and Rank-sum) tests.

### Study status

We have conducted formative interviews with stakeholders (providers, HIV-positive parents, and community members). We began mother-infant enrollment in the study in June of 2017. During the initial 6 months, participant enrollment and full implementation of the pilot were limited by external challenges (prolonged national nurses’ strike and Alere q manufacturer’s cartridge stock-out), which were resolved, setting the stage for normal operations. Participant enrollment is ongoing and expected to conclude in December 2018. We plan to report study results to a national technical working group on implementation of birth and POC testing in Kenya, to the Ministry of Health, and to administrators of the participating facilities. Data will be published in peer-reviewed scientific journals.

## Discussion

The effectiveness of ART in HIV-infected infants is optimal when HIV is diagnosed early, so that treatment can be initiated before a peak in mortality observed between 2 and 3 months [16]. For this reason, Kenyan government guidance issued in 2016 recommends adding birth HIV DNA PCR testing to the standard regimen of PCR tests at 6 weeks, 6 months, and 12 months [19]. However, to date, this is only a provisional recommendation pending pilot data to inform national scale-up. This study will contribute needed pilot data to inform decision making for birth testing with traditional HIV DNA PCR or with POC strategies. Notably, recent data from South Africa suggest uptake of repeat testing at 6 or 10 weeks of age may be diminished among infants already diagnosed HIV-negative at birth [34]. A reduction in retesting rates compromises detection of intrapartum transmissions, thus the impact of birth testing on repeated testing at 6 weeks is an important concern for governments considering policy changes. In addition to assessing uptake and acceptability of the new testing time point and testing strategy (POC), this pilot and feasibility study is designed to assess retest rates for infants tested within the birth window to better understand implications for the Kenyan context.

POC testing also offers potential to improve EID system efficiency in low resource settings where there are multiple delays inherent in the laboratory-based HIV DNA PCR testing process. Sample testing in the clinic setting potentially facilitates mother notification of infant HIV status on the same day as test. To fully realize the POC technology’s potential at the true point of care, cadres of clinical providers must be trained to perform diagnostic procedures outside of traditional clinical duties. Therefore, in addition to traditional laboratory personnel, our pilot study includes training nurses to conduct POC testing within the Maternity and MCH settings. This pilot will solicit provider feedback regarding issues of patient flow, interdepartmental collaboration, and provider workload, while identifying potential barriers to this task-shifting approach during larger scale-up. Other performance measures reflecting the utility of POC platforms—such as user friendliness, equipment durability, test failure rate, costs of maintenance, and frequency of cartridge stockouts—will likely affect the consistency of testing operations and missed opportunities for diagnosis. This pilot study will track these variables and thus supply preliminary feasibility data to inform effective and sustainable POC interventions for larger future studies.

These novel data on implementation outcomes and clinical impact of birth HIV testing with POC and PCR testing strategies are timely given the movement toward more rigorous EID in low resource countries. Implementation and user satisfaction data from the two approved POC platforms, GeneXpert and Alere q, may also guide other groups considering investment in POC scale-up. By providing parameters to estimate effect size and intraclass correlation, these results will facilitate the design of properly powered pragmatic trials to evaluate the impact of birth and POC HIV testing in Kenya. These timely data may also facilitate wider adoption of 2016 EID guidelines issued by WHO and the government of Kenya to accelerate infant testing within the existing PCR infrastructure, while bridging the birth testing strategy to implementation of POC testing technologies.

## Additional file

Additional file 1: Written informed consent: point-of-care testing enrollment. (PDF 49 kb)

## Abbreviations

ART: Antiretroviral therapy
CCC: Comprehensive care center
DBS: Dried blood spot
EID: Early infant diagnosis
HIV: Human immunodeficiency virus
IQR: Interquartile range
MCH: Maternal and child health
PCR: Polymerase chain reaction
PMTCT: Prevention of mother to child transmission
POC: Point-of-care
SD: Standard deviation
SOC: Standard-of-care
TAT: Turnaround time
VL: Viral load
WHO: World Health Organization
