# Supplementary material for: Infant HIV testing at birth using point-of-care and conventional HIV DNA PCR: an implementation feasibility pilot study in Kenya
Source: Pilot Feasibility Stud. 2019 Jan 25;5:18. doi: 10.1186/s40814-019-0402-0 (PMC6347792; doi:10.1186/s40814-019-0402-0)
Supplement: Supplementary file 1 — Written informed consent: point-of-care testing enrollment. (PDF 49 kb) [file 40814_2019_402_MOESM1_ESM.pdf]

APPENDIX I: WRITTEN INFORMED CONSENT: POINT OF CARE TESTING ENROLLMENT

STUDY TITLE:
Piloting at-birth point of care HIV testing strategies in Kenya

Principal Investigators:
Mr. Raphael Lwembe, PhD
Dr. Sarah Finocchario Kessler, PhD, MPH

INTRODUCTION

You have been asked to participate in this research because you are enrolled in Prevention of Mother-to-Child Transmission (PMTCT) services or have an infant who is being tested for HIV. Infant HIV testing is usually conducted through PCR when the infant is 6 weeks old, but results can take a long time to return to the hospital. We would like to test a new point of care (POC) HIV testing system that would allow results for infant HIV testing within hours. In addition to the standard HIV DNA PCR, this test will be performed at birth and when the infant is 6 weeks of age. Earlier diagnosis of HIV and initiation of medication in infants can significantly improve their health and long term outcomes. Your enrollment in PMTCT services and/or your infant's enrollment in early infant diagnosis (EID) services makes you eligible for enrollment in this study.

PURPOSE OF THE STUDY

The proposed study will pilot the new point of care testing strategies for at-birth and 6-week infant HIV testing.

PROCEDURES OF THE STUDY

This study will be carried out at four facilities in Kenya: two of the facilities will use the Alere-Q point of care testing system and two facilities will use the GeneXpert point of care testing system. If you agree to participate, hospital staff will conduct a heel stick to collect two blood samples from your infant at both birth and at 6 weeks of age. The first sample will be for the POC test for cartridge and the second for a dried blood spot sample for HIV DNA PCR, as is standard of care. Results of the POC test will be available within hours. Results of the HIV DNA PCR may take up to 3 weeks or more to be returned to the hospital.

Maternal and infant information will be tracked through the HITSystem, an automated intervention that alerts providers and mothers when PMTCT or EID services are due. We will send you an SMS text messages to remind you when your PMTCT or EID services are needed. While the HITSystem will monitor your and your infant's PMTCT and EID services through completion of EID, data for this research will only be collected until you received the result of your infant's 6 week HIV DNA PCR. If your infant tests HIV-positive we will provide quarterly viral load and CD4 monitoring until your infant reaches 24 months of age.

BENEFITS OF THE STUDY

If you enroll in this study, it means that your infant will benefit from an at-birth point of care HIV test, an at-birth HIV DNA PCR, and a 6 week POC test, in addition to the standard 6 week HIV DNA PCR. For each point of care test, you can be informed of your infant's status the same day as the test was run. Earlier (at-birth) testing and same day results can facilitate earlier initiation of medication for positive infants and improve their long-term health. HIV-positive infants will receive enhanced follow up and monitoring, which will help your doctor make decisions about his/her care.

RISKS OF THE STUDY

There is a risk of stigma and violence in dealing with issues around HIV and each hospital in this study has a group of HIV Testing Counselors (HTC) who are trained to provide HIV-specific social support for issues arising in conjunction with testing and treatment, including intimate partner violence. HTC counselors are part of the established continuum of HIV care at government hospitals and will be available to support mothers dealing with stigma and violence.

We also work closely with the group Mothers to Mothers, who have standardized training and experience with disclosure, PMTCT, and childbearing to provide support and encouragement to HIV+ women enrolled in PMTCT who are new to these issues.

CONFIDENTIALITY

We would like to let you know that we will put in place measures to ensure that your confidentiality as a mother/guardian/infant is protected. This will include the use of numeric codes rather than your name to link your PMTCT/EID related data. The information that you provide to us will be treated with the strictest confidentiality. Only the persons contacting you from the health facility who are trained healthcare workers and the persons directly involved in this study will have access to your information.

CONTACT OF PRINICPAL INVESTIGATOR

You are free to seek clarity or ask any questions at any point in time in the course of the study. If you desire to get furnished with more information concerning the study, feel free to call Raphael Lwembe on Tel number: +254718876976.

CONTACT OF KEMRI SCIENTIFIC AND ETHICAL REVIEW UNIT

Should you have any need or desire to contact KEMRI Scientific and Ethical Review Unit, you may do so using the telephone no. +254(02) 2722541 or by email seru@kemri.org for any queries, at any time of the day during the duration of the study.

COMPENSATION

Participants at certain study sites may be eligible for travel support for their infant's at-birth and 6 week test. Caregivers of HIV-positive infants will receive a stipend of 200 Kenyan shilling for completing an adherence survey at quarterly appointments for their infant's care and treatment (through 2 years).

STORAGE, EXPORTATION OF SAMPLES AND FURTHER STUDIES:

HIV DNA PCR samples utilized for this study will be handled according to national guidelines for EID and will not be subjected to additional storage, exportation or study. POC samples utilized in this study will be processed per the manufacturer's instructions and discarded according to policies established for biospecimens at each of the participating hospitals.

CONSENT AND SIGNATURE OPTIONS

You are free to either participate or not to participate in this study. If you decide you do not want to participate in the study, the testing services will still be offered to you and your baby and you will get the test results using the existing channels at the heath facility for receiving them. You also have a right at any time to change your mind not to participate in the study even after accepting to participate.

CHOOSE OPTION 1 OR 2 BELOW BASED ON YOUR DECISION

1. I have read the information stated above and have had the opportunity to ask questions regarding to the above-mentioned study. I have therefore accepted to participate in the study

Name of Participant:…………………………………….….

Mobile Phone Number:…………………………………

Signature/Thumb Sign:………………………….

Date:………………………………..

Name of trusted healthcare worker:……………………………………….

Mobile Phone number:……………………………………………………..

Date:…………………………………………………………………..

OR

---

2. I have read the information stated above and have had the opportunity to ask questions regarding to the above-mentioned study. I have therefore refused to participate in the study

Name of Participant:……………………………………..….

Signature/Thumb Sign:………………………….

Date:………………………………..

I, the undersigned, have fully explained the relevant details of this study to the person mentioned above. I am qualified to perform the role of Principal Investigator in this study.

Name of Principal Investigator or designated person: ……………………………………………
Name and Role

Signature:……………………

Date:……………………………..

Name of witness:…………………………………………

Signature:…………………..

Date:………………

KIAMBATANISO I: RIDHAA YA HABARI ILIYOANDIKWA: USAJILI WA KUPIMA VIRUSI VYA UKIMWI KATIKA KITUO CHA HUDUMA

JINA LA UTAFITI:
Majaribio ya Utekelezaji wa uhakika wa huduma ya kupima virusi vya ukimwi wakati mtoto muathiriwa anapozaliwa.

Watafiti wakuu:
Dr. Raphael Mwamtsi Lwembe, PhD
Dr. Sarah Finocchario Kessler, PhD, MPH

UTANGULIZI

Umealikwa kushiriki utafiti huu kwa sababu umesajiliwa kwenye mfumo wa huduma ya kuzuia uambukizaji wa ukimwi kutoka kwa mama hadi kwa mtoto au uko na mtoto mchanga ambaye anapata huduma za kuambatana na virusi vya ukimwi. Kwa kawaida, virusi vya ukimwi vinatambuliwa kutumia mbinu ya kisasa inayoitwa PCR wakati mtoto akiwa umri wa wiki sita, ingawa matokeo ya mbinu hii inaweza kuchukua muda mrefu kufika kwa kituo cha matibabu. Tungelipenda kutafiti mfumo mpya wa kupima virusi vya ukimwi katika maeneo ya huduma ambao utawezesha matokeo kwa masaa machache. Kuongezea tekinolojia hii ya kiwango inayoitwa DNA PCR, mfumo huu mpya tunaonuwia kutafiti utatekelezwa mara tu mtoto anapozaliwa na pia mtoto anapofika umri wa wiki 6. Kutambulika mapema kwa virusi vya ukimwi huelekea uanzishwaji wa madawa ya virusi vya ukimwi kwa watoto wachanga, hali ambayo inaweza kuboresha afya na matokeo mazuri ya kiafya ya baadae. Kwa sababu umesajiliwa kwa huduma ya PMTCT na/au mtoto wako amesajiliwa kwa huduma ya EID, unahitimu kushiriki katika utafiti huu.

MADHUMUNI YA UTAFITI.

Utafiti huu unaopendekeza kujaribia mbinu mpya ya utekelezaji wa mikakati inayopendekezwa inchini ya kupima virusi vya ukimwi katika vituo vya huduma wakati mtoto mchanga anapozaliwa na pia mtoto akiwa umri wa wiki 6.

TARATIBU ZA UTAFITI

Utafiti huu utafanyika katika vituo vya afya vi nne inchini Kenya; vituo viwili vitatumia tekinolojia ya Alere-Q na vituo vingine viwili vitatumia tekinolojia ya GeneXpert.

Ukikubali kushiriki utafiti huu, wahudumu wa hospitali watatoa sampuli ya damu kutoka kwa mtoto mchanga wakati wa kuzaliwa na akifikisha umri wa wiki 6 kutumia mbinu fulani inayohusisha utoaji wa damu kupitia kisigino cha mtoto ijulikanayo kwa lugha ya kiingereza kama “heel stick” . Sampuli ya kwanza ya damu itatumiwa kwa mbinu mpya tunayoitafiti ili hali sampuli ya damu ya pili itakaushwa na kutumiwa kwa mbinu yenye kwa sasa inatumiwa na kukubalika inayoitwa HIV DNA PCR. Matokeo ya mbinu ya utafiti yatapatikana ndani ya masaa machache. Matokeo ya DNA PCR yanaweza kutokea baada ya hata wiki tatu au Zaidi.

Habari za mama na mtoto zitawasilishwa kupitia uvumbuzi mpya unaoitwa HITsystem, mfumo unaotuma tahadhari kwa wahudumu na kina mama wakati wanapohitajika kwa huduma za PMTCT au EID. Ili hali uvumbuzi wa HITSystem utasaidia kufuatilia huduma za mama na mtoto kwa PMTCT na EID, ujumbe na ufahamu utakaopatikana kwa utafiti huu utakusanywa na kuwekwa pamoja hadi matokeo ya kupima virusi vya ukimwi kutumia DNA PCR katika umri wa wiki 6 yatakapotokea.

FAIDA ZA UTAFITI.

Ukikubali kushiriki katika utafiti huu, mtoto wako mchanga atanufaika na mfumo wetu tunaonuwia kuutafiti wa kupima virusi vya ukimwi wakati wa kuzaliwa kwenye kituo cha huduma, pia atapata matokeo ya tekinolojia ya DNA PCR wakati wa kuzaliwa na pia wiki 6 baada ya kuzaliwa.Matokeo ya virusi vya ukimwi ya mtoto

mchanga itajulikana kwa siku moja. Matokeo ya siku ya kuzaliwa yatasaidia sana kuanzisha matibabu kwa wale watoto wanaozaliwa na virusi vya ukimwi ili kuboresha hali yao ya afya. Iwapo mtoto wako mchanga atapatikana ameambukizwa na virusi vya ukimwi basi tutafuaatilia vipimo vya kiwango cha virusi vilivyo kwenye damu na pia chembechembe za kinga za damu aina ya CD4 hadi mtoto wako mchanga atakapofikisha umri wa miezi 24.

HATARI ZA UTAFITI.

Kuna hatari ya athari ya unyanyapaa na vurugu wakati wa kuhusika na maswala ya ukimwi na kila hospitali katika utafiti huu iko na kikundi cha washauri wa maswala ya ukimwi, waliopokea mafundisho ya kupeana huduma na msaada spesheli za kijamii za virusi vya ukimwi ikiwemo kupima na kuanzisha matibabu, pamoja na vurugu kati ya mume na mke kwa jamii. Ushauri ni moja ya huduma inayotolewa kwenye vituo vya huduma za ukimwi katika hospitali za serikali na washauri watakuwepo kuwasaidia kina mama kupigana na unyanyapaa na vurugu za kijamii.

Pia tutafanya kazi karibu na kikundi cha kina mama, ambao wameratibu mafunzo na uzoefu kwa mambo ya kujitambulisha hali ya virusi vya ukimwi, PMTCT, na uzalishaji ili kuwasaidia na kuwapea moyo wanawake walio na virusi vya ukimwi waliosajiliwa kwa huduma ya PMTCT na ambao ni wageni kwa maswala ya virusi vya ukimwi. Watoto wachanga walioambukizwa na virusi vya ukimwi watafuatiliwa kwa hali ya kuimarika, hali ambayo itasaidia dakitari kufanya maamuzi kuhusu huduma za matibabu za watoto hao.

USIRI.

Tungelipenda kukufahamisha kwamba tutaweka hatua ili kuhakikisha kwamba usiri wako umehifadhiwa. Hatua hizi ni pamoja na kutumia vificho kuambatana na ujumbe husika, badala ya kutumia majina. Ujumbe utakaopeana utatunzwa kwa njia ya faragha na usiri. Wahudumu wale watakaowasiliana na wewe kutoka vituo vya huduma pekee, waliopokea mafunzo ya huduma za kiafya, na wale wanaohusika na utafiti huu moja kwa moja ndio wataruhusiwa kukaribia ujumbe wako.

MAWASILIANO NA MTAFITI MKUU

Uko na uhuru wa kutafuta uwazi ama kuuliza maswali wakati wowote utafiti huu ukiendelea. Iwapo utapendelea kupata maelezo zaidi kuhusu utafiti huu, jisikie huru kupiga simu kwa Dakitari Raphael Lwembe kwa nambari: +254 (0) 718876976

MAWASILIANO NA KITENGO CHA KEMRI KINACHOHUSIKA NA KUANGALIA MAADILI YA UTAFITI WA KISAYANSI

Iwapo utakuwa na uhitaji au hamu ya kuwasiliana na kitengo cha KEMRI kinacho shughulikia maswala ya maadili ya utafiti wa kisayansi, unaweza kufanya hivyo kupitia nambari ya simu +254 (02) 2722541 au kwa barau pepe seru@kemri.org kwa maswali yoyote siku yoyote katika kipindi cha utafiti huu.

FIDIA

Washiriki katika hospitali fulani inaweza kuwa na haki kwa ajili ya msaada wa usafiri kwa watoto wao wa mtihani katika kuzaliwa na 6 wiki. Walezi wa watoto wachanga wenye VVU kupokea kiasi kidogo cha fedha za 200 Kes kwa ajili ya kukamilisha utafiti kuzingatia katika uteuzi baada ya miezi mitatu kwa kuhudumia mtoto mchanga wao na matibabu (hadi miaka 2).

UHIFADHI, USAFIRISHAJI WA SAMPULI YA DAMU KWA MASOMO YA ZIADA

Sampuli za damu zitakazotumiwa kwa mbinu ya HIV DNA PCR zitatumika kulingana na kuambatana na maadili ya EID wala hakutakuwa na uwekaji wa sampuli hizi za damu ama usafirishaji nje ya nchi. Sampuli za damu zitakazotumiwa kwa mbinu mpya tutakayoitafiti zitatayarishwa kulingana na maagizo ya kampuni zilizobuni tekinolojia hizi na kulingana na kuambatana na kanuni zilizowekwa za kutafiti sampuli kama hizo.

IDHINI NA SAHIHI BADALA

Uko na uhuru wa kushiriki au kutoshiriki katika utafiti huu. Iwapo hujiskii kushiriki utafiti huu, hakutakuwa na athari zezote kwa ubora wa huduma unazopewa wewe na mtoto wako kwa sasa na utapata matokeo kutumia mbinu zinazotumika tayari kwa sasa. Uko na haki wakati wowote kubadili nia yako kutoshiriki kwa utafiti huu hata baada ya kukubali.

CHAGUA CHAGUO 1 AU 2 LINALOFUATA KWA MSINGI WA UAMUZI WAKO

1. Nimesoma habari iliyoelezwa hapo juu na nimepata nafasi ya kuuliza maswali kuhusu utafiti huu. Kwa hiyo nimekubali kushiriki utafiti huu.

Jina la Mshiriki: ....

Nambari ya Simu ya Mkono: ....

Sahihi/ Alama ya kidole gumba: ....

Tarehe: ....

Jina la mhudumu wa afya unaye muamini: ....

Nambari yake ya simu ya Mkono: ....

Tarehe: ....

AU

---

2. Nimesoma habari iliyoelezwa hapo juu na kupata nafasi ya kuuliza maswali kuhusiana na utafiti huu. Kwa hiyo nakataa kuhusika na utafiti huu:

Jina la Mshiriki aliyekataa: ....

Sahihi/ Alama ya kidole gumba: ....

Tarehe: ....

Mimi niliyetia sahihi nimetoa maelezo ya kutosha kuhusiana na utafiti huu, kwa aliye tajwa hapo juu. Niko na tajriba ya kutekeleza majukumu ya Mpelelezi Mkuu wa utafiti huu.

Jina la Mpelelezi Mkuu au Muidhinishwa: ....

Jina na Jukumu

Sahihi: ....

Tarehe: ....

Jina la shahidi: ....

Sahihi: ....

Tarehe: ....
